# Validation of two nurse-based screening tools for delirium in elderly patients in general medical wards

**DOI:** 10.1186/s12912-020-00464-4

**Published:** 2020-07-31

**Authors:** Manuela Bergjan, Max Zilezinski, Torsten Schwalbach, Christiana Franke, Hebun Erdur, Heinrich Jakob Audebert, Armin Hauß

**Affiliations:** 1 Charité – Universitätsmedizin Berlin, corporate member of Freie Universität Berlin, Humboldt-Universität zu Berlin and Berlin Institute of Health, Business Division Nursing Directorate – Nursing Science, Charité – Universitaetsmedizin Berlin, Chariteplatz 1, Berlin, 10117 Germany; 2 Charité – Universitätsmedizin Berlin, corporate member of Freie Universität Berlin, Humboldt-Universität zu Berlin and Berlin Institute of Health ,Business Division Nursing Directorate – Nursing Science, Core-Team III Delirium Management and Dementia Care, Charité – Universitaetsmedizin Berlin, Charitéplatz 1, 10117 Berlin, Germany; 3 Charité – Universitätsmedizin Berlin, corporate member of Freie Universität Berlin, Humboldt-Universität zu Berlin and Berlin Institute of Health, Department of Neurology with Experimental Neurology Campus Benjamin Franklin (CBF), Charité – Universitaetsmedizin Berlin, Hindenburgdamm 30, Berlin, 12200 Germany

**Keywords:** Delirium, Screening, Nursing delirium screening Scale, Delirium observation screening Scale, Risk factor, Confusion

## Abstract

**Background:**

Delirium is an acute disturbance characterized by fluctuating symptoms related to attention, awareness and recognition. Especially for elderly patients, delirium is frequently associated with high hospital costs and resource consumption, worse functional deterioration and increased mortality rates. Early recognition of risk factors and delirium symptoms enables medical staff to prevent or treat negative effects. Most studies examining screening instruments for delirium were conducted in intensive care units and surgical wards, and rarely in general medical wards. The aim of the study is to validate the Nursing Delirium Screening Scale (Nu-DESC) and the Delirium Observation Screening Scale (DOS) in general medical wards in a German tertiary care hospital, considering predisposing delirium risk factors in patients aged 65 and older.

**Methods:**

The prospective observational study including 698 patients was conducted between May and August 2018 in two neurological and one cardiology ward. During their shifts, trained nurses assessed all patients aged 65 or older for delirium symptoms using the Nu-DESC and the DOS. Delirium was diagnosed according to the DSM-5 criteria by neurologists. Patient characteristics and predisposing risk factors were obtained from the digital patient management system. Descriptive and bivariate statistics were computed.

**Results:**

The study determined an overall delirium occurrence rate of 9.0%. Regarding the DOS, sensitivity was 0.94, specificity 0.86, PPV 0.40 NPV 0.99 and regarding the Nu-DESC, sensitivity was 0.98, specificity 0.87, PPV 0.43, NPV 1.00. Several predisposing risk factors increased the probability of delirium: pressure ulcer risk OR: 17.3; falls risk OR: 14.0; immobility OR: 12.7; dementia OR: 5.38.

**Conclusions:**

Both screening instruments provided high accuracy for delirium detection in general medical wards. The Nu-DESC proved to be an efficient delirium screening tool that can be integrated into routine patient care. According to the study results, pressure ulcer risk, falls risk, and immobility were risk factors triggering delirium in most cases. Impaired mobility, as common risk factor of the before mentioned risks, is well known to be preventable through physical activity programmes.

## Background

Delirium is an acute disturbance characterized by fluctuating symptoms related to attention, awareness, and recognition [1]. Especially elderly people afflicted with pre-existing cognitive impairment, delirium is associated with poor outcomes, i.e. functional decline, admission to long-term care, increased risk of falling, increased duration of hospital stay and higher mortality rates [2–4]. Moreover, delirium increases hospital costs [2–4]. Delirium often go undiagnosed and undiscovered [5–7]. Studies report the prevalence of delirium in different hospital settings to be in the range between 8 and 50% and the incidence to be between 10 and 82% [2, 8–11]. The highest overall occurrence rates of delirium are generally reported from intensive care units (7–82%), followed by palliative care units (6–74%) [12], surgical wards (11–51%), and general medical wards (11–35%) [2].

The most significant predisposing risk factors for delirium are age (65 years or older), cognitive impairment and severity of illness [2, 13]. For hearing and vision impairment exits contradictory results [13–15]. Other predisposing risk factors, e.g. care dependency and immobility, which are of high relevance from a nursing perspective, have rarely been studied to date [13, 16, 17]. Precipitating risk factors include infection, polypharmacy, pathological laboratory findings, and physical restraint [14, 15, 18]. Looking at all these factors, it is not surprising that delirium is a common ailment of especially elderly hospitalized patients.

International guidelines recommend delirium screening employing valid and reliable instruments appropriate for the specific setting for at-risk patients [19, 20]. There are many delirium-screening tools using three different types of instruments: observation, interviews and tests, and combinations of these instruments [7, 21].

Because nurses spend most of their work-time in direct patient care, they are in a prominent position to observe changes in the behaviour of their patients during the shift. Only a few observational instruments, which have been validated and proven reliable, are available in German [7]. The Nursing Delirium Screening Scale (Nu-DESC) was translated and validated by Luetz et al. [22] and Radtke et al. [23, 24] in different postoperative hospital settings. The Delirium Observation Screening Scale (DOS) was also translated [25] and validated in German [26].

Stringent psychometric properties are important requirements for screening tools. For both scales, studies have been published for different settings, e. g. intensive care, recovery room, cardiac surgery [22–24, 27–30]. A research gap was identified for the Nu-DESC and the DOS regarding general medical wards in German hospitals. Therefore, Table 1 provides an overview of the results of several studies.

**Table 1:** Test Characteristics of the Nu-DESC and DOS in general wards

Instrument	Authors	n	Setting	Cut-Off	Sensitivity	Specificity	PPV	NPV
Nu-DESC	Gaudreau et al. 2005 [31]	146	Hemato-oncology, Internal medicine	≥2	0.86	0.87	no data	no data
Nu-DESC	Leung et al. 2008 [32]	100	Geriatric rehabilitation	≥1	0.96	0.79	no data	no data
Nu-DESC	Hargrave et al. 2017 [33]	405	Neurology, Neurosurgery, General medicine, General or orthopaedic surgery	≥1	0.67	0.93	0.74	0.92
Nu-DESC	Spedale et al. 2017 [34]	101	Nephrology, (Ortho)-Geriatrics, Neurology, Cardiology, Pulmonology	≥2	0.64	1.00	0.36	1.00
DOS	Van Gemert & Schuurmans et al. 2007 [35]	86	General medical and surgical	≥3	0.89	0.88	0.47	0.99
DOS	Detroyer et al. 2014 [36]	45	Palliative care	≥3	0.82	0.96	0.69	0.98
DOS	Gavinski et al. 2016 [37]	54	General medical	≥3	0.90	0.91	0.53	0.99
DOS	Hasemann et al. 2018 [26]	104	General medical	≥3	0.56	0.92	0.73	0.83

*DOS* Delirium Observation Screening Scale, *NPV* negative predictive value, *Nu-DESC* Nursing Delirium Screening Scale, *PPV* positive predictive value

Studies have reported a wide range of sensitivity of the Nu-DESC, depending on the selected cut-off point while the sensitivity of the DOS has generally been reported as high [35–37]. Due to false positive results, PPV is moderate for both tools [33–37]. NPV and specificity values [33–37] of both tools are high.

In addition to the psychometric properties, nurses need a feasible screening tool for daily clinical care. Studies report that the time needed to test patients for delirium using the five-item Nu-DESC, amounts to one to two minutes while the 13-item DOS, requires five minutes [31, 35].

The aim of our study was to validate the Nu-DESC and the DOS in general medical wards in our tertiary care hospital with consideration of predisposing risk factors for delirium for patients aged 65 and older. This prospective observational study aimed at determining which of the instruments was the better alternative to be employed at our tertiary care hospital.

## Methods

The prospective observational study was conducted between May and August 2018 in two neurological and one cardiology wards and included patients aged 65 or older, regardless of whether they suffered from dementia upon admission. Data were collected from admission to discharge, up to a maximum of 20 days. Patients at the end of life were excluded from the study for ethical reasons.

Within six hours after admission of the patients, bedside nurses assessed the individual pressure ulcer risk, risk of falling, immobility, hearing and vision impairment and documented the findings in an electronic patient record.

Pressure ulcer risk and falls risk were measured according to the risk factors recommended by the German expert standards in nursing and international guidelines [38–42]. The German expert standards did not explicit recommend valid and reliable instruments like for example the Braden, Norton or Waterlow Scale for pressure ulcer risk or the Morse Score, Hendrich Fall Risk Model or STRATIFY for falls risk. They recommend clinical assessment taking into account the risk factors relevant for the respective setting, a valid and reliable instrument can be used as support. Pressure ulcer risk factors include impaired mobility (ability to change and control body position, e.g. completely immobile, very limited), impaired activity (degree of physical activity, e.g. bedfast, chairfast), existing pressure ulcer, and medical devices. Falls risk factors include impaired mobility and/or activity, falls over the last twelve months, impaired continence, impaired cognition and polypharmacy. Immobility as a common risk factor for pressure ulcer and falls risk was defined as being unable to performed frequent or significant positional changes independently in bed. Dementia diagnosis, cause of admission according to the ICD-10 classification system [43] and polypharmacy were retrieved from medical records. Polypharmacy was defined as the concurrent use of more than seven drugs.

Gaudreau et al. (2005) developed the Nu-DESC for professional nurses. It was first psychometrically tested in an oncological acute care setting [31]. The Nu-DESC has been translated into many languages [28, 29, 32, 34, 44, 45]. The Nu-DESC assesses disorientation, inappropriate behaviour, inappropriate communication, illusions/ hallucination, and psychomotor retardation. Each of the five items is rated from on a scale ranging from zero (no symptoms) to two (strong symptoms), resulting in a total score from zero to at most ten. The cut-off for delirium detection using the Nu-DESC is reported to be two in most cases.

Schuurmans et al. developed the Delirium Observation Screening Scale in early 2000 as a method for screening for delirium based on nurses’ observations made during the inpatient stay [46, 47]. Initially, the Delirium Observation Screening Scale included 25 items. In a follow-up study, the DOS was reduced to 13 items. A validated German version of the DOS is available [25, 26]. Each of the items is rated either as zero (normal behaviours), one (sometimes or often) or not assessable. The total score varies from 0 to 13. The cut-off value of the DOS is three points. The DOS has been translated into a few languages [36, 37, 46].

The nurses were systematically trained and supervised by nursing experts for delirium care and management. On each ward, three training sessions of 45 min each were offered.

The Nu-DESC and the DOS scores were obtained by bedside nurses during every shift (3 times per day). If one or both of the screenings tools scored positive, patients were assessed by neurologists for delirium diagnosis (hypoactive, hyperactive, and mixed form) within 48 h based on the gold standard DSM-5 criteria, using detailed medical history, interviews with patients, proxies, nurses and/or physicians. In addition, the 3-Minute Diagnostic Confusion Assessment Method (3D-CAM) [48] was used by the neurologists. For all patients featuring score below the cut-off points, the nursing experts performed a structured examination of the medical charts using the DSM-5 criteria.

### Statistical methods

Data were tabulated in Microsoft Excel (R) for overview purposes descriptive statistics were computed for all variables of the study. Discrete variables were expressed as counts / percentage (pressure ulcer risk, falls risk, immobility, hearing, vision impairment, dementia, sex). The sample was separated according to delirium status (delirium / no delirium). Therefore, Fisher’s exact test was used for sex and all predisposing risk factors (Table 7) in this study. We considered a *P*-Value below 0.05 as being significant. The odds ratio (OR) was calculated for the predisposing risk factors with a Confidence Interval of 95% (CI). Continuous variables (age, length of stay) as medians with 25- and 75 percentiles, because these factors were not normally distributed. Differences between both groups were assessed using the Mann-Whitney U-test. Diagnostic performance of the Nu-DESC and DOS was evaluated. The anticipated effect sizes and optimal sample size for the current study were calculated on the base of the data (sensitivity/ specificity) reported in a systematic review and previous studies [7, 23, 46]. The comparison between the delirium assessment methods was bivariate and was analysed using the McNemar test. Sensitivity, specificity, positive predictive values and negative predictive value were calculated. Characteristics of the diagnostic test were visualized in a receiver operating characteristic (ROC) curve. The statistical analysis was performed using IBM SPSS Statistics 25.

## Results

In total 745 patients, aged 65 and older were screened by nurses during the three- month period. Reasons for exclusion of 47 patients were duration of stay less than 24 h, no clinical consultation possible, or missing dataset. Six hundred and ninety-eight patients were included in the analysis. The neurologists assessed 165 patients. From these patients *n* = 133 showed positive Nu-DESC and DOS rates, *n* = 15 only positive DOS rates and *n* = 12 only positive Nu-DESC rates. Five patients were evaluated by the neurologist with negative ratings (one point under each of the cut offs) in both screening instruments.

### Patient characteristics

Differences in basic characteristics of patients with and without delirium are depicted in Tables 2 and 3. Patients without delirium were significantly younger (*p* < 0.001). Additionally, the duration of the stay in the medical units was longer for the group with delirium (*p* < 0.001).

**Table 2:** Basic patient characteristics

	Delirium DSM-5 (*n* = 63)	No-Delirium DSM-5 (*n* = 635)	*P* Value
Sex [1]			0.693
male	33	314	
female	30	321	
Age in years [2]	82 (78–87)	77 (72–82)	< 0.001
Duration of stay: medical wards (days) [2]	5 (3.5–7.5)	3 (2–6)	< 0.001

[1] Absolute values, *p* values for the exact Fisher’s test

[2] Median, percentiles in brackets, p values for Mann-Whitney U test

**Table 3:** Cause of admission according to ICD-10 classification

Cause of admission (ICD-10 Classification)	Delirium DSM-5 (n = 63)	No-Delirium DSM-5 (***n*** = 635)
Certain infectious and parasitic diseases (A00-B99)	2 (3.2%)	2 (0.3%)
Diseases of the nervous system (G00-G99)	10 (15.9%)	127 (20.0%)
Diseases of the circulatory system (I00-I99)	46 (73.0%)	383 (60.3%)
Diseases of the respiratory system (J00-J99)	2 (3.2%)	10 (1.6%)
Symptoms, signs and abnormal clinical and laboratory findings, not elsewhere classified (R00-R99)	2 (3.2%)	48 (7.6%)
Injury, poisoning and certain other consequences of external causes (S00-T98)	1 (1.6%)	3 (0.5%)
Other diagnosis	0 (0.0%)	62 (9.8%)

Most of the patients were admitted with diseases of the circulatory system (J00-J99) with the largest group being patients suffering from patients with cerebrovascular diseases (I63-I69) *n* = 184 (26.4%). 151 (21.6%) of the patient had an admitting cause of any other forms of heart disease (I30–52) and 85 (12.2%) Ischemic heart diseases (I20-I25) and nine (1.3%) with other diseases of the circulatory system.

The other diagnosis are subdivided in diseases of General symptoms and signs, Polyneuropathies and other disorders of the peripheral nervous system, Symptoms and signs involving cognition, perception, emotional state and behaviour, Persons encountering health services for specific procedures and health care, Extrapyramidal and movement disorders, diseases of the eye and adnexa, diseases of the nervous system, symptoms, signs and abnormal clinical and laboratory findings, not elsewhere classified and diseases of respiratory system.

### Comparison of test performance

Sensitivity and specificity were high in both instruments as shown in Table 4. Fifty-nine (94%) patients with verified delirium were tested positive for delirium by the DOS and 62 (98%) by the Nu-DESC. The DOS scored false positive in 14% of the cases, the Nu-DESC in 13%. PPV was low in both instruments due to the amount of false positive ratings. Of the 635 patients without delirium, 89 were identified as being false positive using the DOS, and 83 using the Nu-DESC.

**Table 4:** Overall Sensitivity, Specificity, PPV and NPV of the two tools

	Total (*n* = 698)	Cardiology (*n* = 307)	Neurology (*n* = 391)
**DOS**			
Sensitivity	0.94 (*n* = 59/ 63)	1.00 (*n* = 14/ 14)	0.92 (*n* = 45/ 49)
Specificity	0.86 (*n* = 546/ 635)	0.95 (*n* = 278/ 293)	0.78 (*n* = 268/ 342)
PPV	0.40 (n = 59/ 148)	0.48 (n = 14/ 29)	0.38 (n = 45/ 119)
NPV	0.99 (n = 546/ 550)	1.00 (n = 278/ 278)	0.99 (n = 268/ 272)
**Nu-DESC**			
Sensitivity	0.98 (*n* = 62/ 63)	1.00 (n = 14/ 14)	0.98 (*n* = 48/ 49)
Specificity	0.87 (*n* = 552/ 635)	0.97 (*n* = 284/ 293)	0.78 (n = 268/ 342)
PPV	0.43 (n = 62/ 145)	0.61 (n = 14/ 23)	0.39 (n = 48/ 122)
NPV	1.00 (n = 552/ 553)	1.00 (n = 284/ 284)	1.00 (n = 268/ 269)

*PPV* Positive Predictive Value, *NPV* Negative Predictive Value

There were differences in specificity between cardiological and neurological patients. The NPV was high for cardiology in both instruments while the Nu-DESC rendered higher scores than the DOS in the neurological ward. The PPV was moderate in both instruments.

**Fig. 1:**
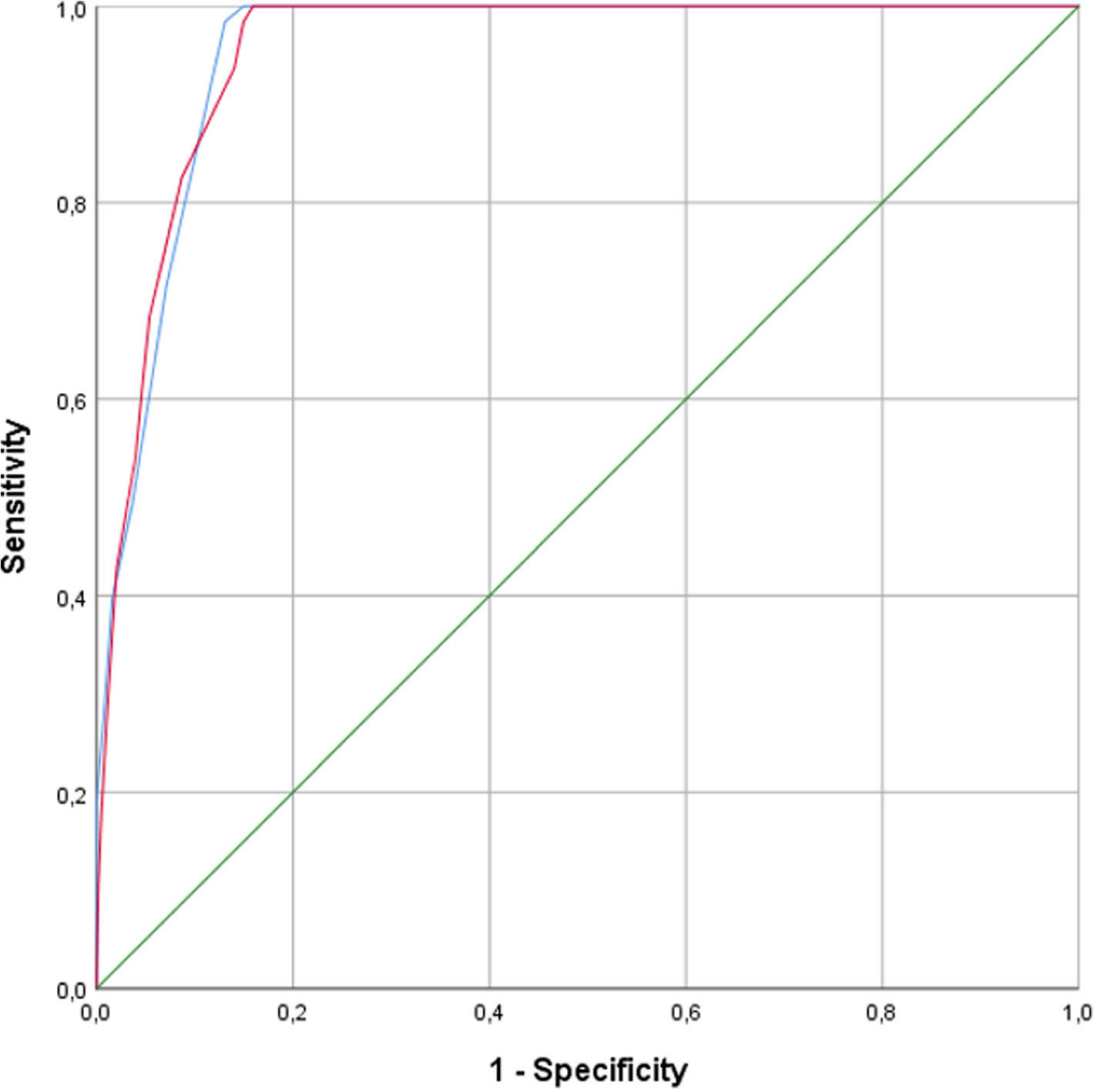
Receiver operating characteristic (ROC). Nu-DESC blue line. DOS red line. Reference line green

The accuracy of the Nu-DESC and DOS was analysed in terms of receiver operating characteristics. The original cut-off points of two and three for Nu-DESC and the DOS, respectively, were confirmed. The area under curve (AUC) is similar for both screening tools, as shown in Table 5 and Fig. 1.

**Table 5:** Area under the curve (AUC)

	AUC	95% CI	*P* Value
DOS	0.955	0.94–0.97	< 0.001
Nu-DESC	0.955	0.94–0.97	< 0.001

### Delirium subtypes

Sixty-three of the 698 patients (9.02%) suffered from delirium either according to DSM-5 criteria and/or the neurological assessment. Table 6 describes the different forms of delirium.

**Table 6:** Type of delirium

Characteristics of patients with delirium	Total	Cardiology	Neurology
Overall occurrence rate of delirium	9.0%	4.6%	12.5%
Overall	63 (100%)	14 (22.2%)	49 (77.8%)
Hyperactive	17 (27.0%)	5 (7.9%)	12 (19.1%)
Hypoactive	7 (11.1%)	0 (0%)	7 (11.1%)
Mixed form	39 (61.9%)	9 (14.3%)	30 (47.6%)

### Predisposing risk factors for delirium

According to the aim of the study, Table 7 shows the data collection of selected predisposing risk factors for delirium, divided into patients with and without delirium.

**Table 7:** Predisposing risk factors for delirium

	n	Delirium DSM 5	n	No-Delirium DSM-5	*P* Value	Odds Ratio (95% CI)
Pressure ulcer risk*	62	57 (91.9%)	582	231 (39.7%)	< 0.001	17.32 (6.84–43.86)
Falls risk*	62	59 (95.2%)	582	339 (58.2%)	< 0.001	14.01 (4.36–45.49)
Immobility*	62	35 (56.5%)	582	54 (9.3%)	< 0.001	12.67 (7.13–22.52)
Dementia*	63	14 (22.2%)	635	32 (5.0%)	< 0.001	5.38 (2.69–10.76)
Hearing Impairment*	56	6 (10.7%)	597	61 (10.2%)	0.82	1.05 (0.43–2.56)
Vision Impairment*	56	13 (23.2%)	594	238 (40.1%)	0.014	0.45 (0.23–0.859)

* *P*-Values for the exact Fisher’s test: in both groups, there are some missing of information’s

Patients known to suffer from dementia had a significantly higher risk of developing delirium during hospitalization. Patients with relevant mobility problems (falls risk/ pressure ulcer risk/ immobility) were more frequently represented in the group of delirious patients. There was also a significant increase in the risk of delirium for patients with polypharmacy using more than seven drugs (*p* = 0.001, OR: 2.63 (1.53–4.49).

## Discussion

Important result of this study was that the German versions of the Nu-DESC and the DOS were confirmed to be valid screening tools for detection delirium in medical patients without surgery in general wards, as both tests provide high sensitivity. The psychometric properties of both screening tools are very similar. Pressure ulcer risk, falls risk, and immobility proved to be relevant predisposing risk factors in this study. Impaired mobility as a common risk factor is potentially modifiable to prevent delirium.

In this study, the Nu-DESC proved to be a sensitive and specific screening tool, although the DSM-5 criteria of delirium ‘*disturbance in attention’* is missing in the score. In comparison to other studies pertaining to the Nu-DESC [33, 34] and the DOS [35–37], this study rendered higher results in terms of sensitivity and specificity. However, both tools are screening tools and requite a comprehensive assessment in collaboration with physicians to diagnose delirium or another diagnosis for patients with positive scores.

Examination of the results from the cardiologic ward reveals, the PPV of the Nu-DESC and DOS to be similar to those obtained by other studies [34, 35, 37]. In Neurology, the low PPV is within the range reported by Spedale et al. (2017) [34], but lower than the one reported by Hargrave et al. (2017) [33]. Both studies described clearly lower sensitivity values. The relatively high rate of false positive screenings, especially in neurological patients, may be related to pre-existing disease, e.g. example stroke, aphasia, dementia, which were wrongly identified as a delirium by the nurse screening the patient specific education programs may result in more accurate scoring rates. The NPV (in neurology and cardiology) of both screening tools were very high and the results were similar to those obtained by other studies [33–35, 37].

Acceptability and feasibility are also relevant quality properties of a screening tool. The five-item Nu-DESC as opposed to the 13-items DOS proved to be superior in terms of, staff acceptance and manageability and was, therefore, favoured.

The sample was homogenous and included a substantial number of neurological and cardiological patients, and was therefore limited comparable to samples used in previous research on the Nu-DESC [31] and the DOS [35] listed in Table 1. In total, 9.0% of the patients suffered from diagnosed delirium according to the gold standard (DSM-5). Although the predisposing risk factors and the cause of admission of the patients included in the study was analysed, the reason for the low overall occurrence rate was impossible to determine. Possible reasons for this phenomenon may include the wide range of inclusion criteria.

Inouye [2] provided an overview of the differences in the overall occurrence rate of delirium between medical populations. In this study, the rate of delirium in the neurology ward was three times higher than the one determined for the cardiology ward. This phenomenon may be explained by the high number of patients with cerebral infarction (23.5%; *n* = 164) in the neurology wards.

The results of this study suggest that pre-existing dementia (OR 5.38) is an important risk factor for delirium, comparable to other studies (OR 2,3–5,2) [18, 49]. For medical inpatients scarce data have been published for falls risk, pressure ulcer risk and immobility [18]. In this study, these risks proved to be statistically significant and linked with high odds ratios. Impaired mobility, as common risk factor of the before mentioned risks, is well known to be preventable through physical activity programmes [50, 51].

In addition, polypharmacy proved to be a relevant clinical risk factor if it refers to the concurrent use of at least seven drugs. As other studies defined polypharmacy differently, comparison of the results is complicated. With polypharmacy being defined as the concurrent use of at least seven a day, the results obtained by this study rendered an OR 2.63, which was in the same range of the results obtained by Hein et al. [52] and Ranhoff et al. [53] (OR between 1.9 and 2.33).

### Limitation and strength

Data of predisposing risk factors were collected, standardized and structured in accordance with the corresponding German and international guidelines. The cohort consisted of neurology and cardiology patients aged 65 and older, thus limiting generalization of the findings to this patient population.

For reasons of feasibility, patients were diagnosed during day shifts from Monday through Friday instead of receiving a diagnostic workup by a neurologist immediately after the patients screened positive for delirium. The medical charts of negatively screened patients were reviewed to confirm the screening results. Therefore, it is possible that some negatively screened patients will have been false negatives.

A strength of this study is that trained ward nursing staff, who cared for the patients during the shift, did the screenings 24 h a day at the end of every early, late and night shift.

## Conclusion

The German versions of both screening instruments Nu-DESC and DOS can be recommended for application in medical wards, as both tools provide high sensitivity and specificity rates. An advantage of the Nu-DESC is that only five items have to be determined in comparison to the thirteen items of the DOS. The Nu-DESC is an efficient delirium screening tool that can be integrated into routine patient care, despite the fact that it lacks an item regarding a disturbance in attention.

Patients with cognitive impairment require additional assessments in order to differentiate delirium from dementia and other diagnoses. Further research is needed for the Nu-DESC and DOS for specific patient populations on general medical wards and the importance for impaired mobility as risk factor.

## Data Availability

The datasets generated and analyzed during the current study are not publicly available, as the authors do not have permission from participants to publish the collected raw data but data can be made available from the corresponding author with permission from the first author Manuela Bergjan on reasonable request.

## Abbreviations

Nu-DESC: Nursing Delirium Screening Scale
DOS: Delirium Oberservation Screening Scale
PPV: Positive predictive value
NPV: Negative predictive value
AUC: Area under curve
ROC: Receiver operating characteristic
OR: Odds ratio
DSM-5: Diagnostic and Statistical Manual of Mental Disorders - 5
ICD-10: International Statistical Classification of Diseases and Related Health Problems - 10
e.g.: Exempli gratia = for example
CI: Confidence Interval
i.e.: id est. = that is
3d-CAM: 3-Minute Diagnostic assessment for Confusion Assessment Method-defined delirium
